# PEEP in SBTs: breathing and beyond

**DOI:** 10.1186/s13613-025-01542-z

**Published:** 2025-08-13

**Authors:** Ichita Yamamoto, Yasuhiro Norisue

**Affiliations:** Department of Emergency and Critical Care Medicine, Tokyo Bay Urayasu Ichikawa Medical Center, 3-4-32 Todaijima, Urayasu, Chiba Japan

Dear Editor,

We read with great interest the editorial by Thille and colleagues, which highlights the limited evidence supporting the use of PEEP during spontaneous breathing trials (SBTs) [1]. While the editorial effectively summarizes recent evidence supporting the use of PSV—with or without PEEP—for SBTs, we believe it also opens the door to a broader discussion on the physiological role of SBTs, especially in high-risk patients.

SBTs were originally developed to physiologically evaluate whether a patient is truly ready for extubation. However, in recent trials and meta-analyses, the focus has shifted toward a different clinical question: which SBT setting statistically lowers the overall reintubation rate? This shift risks obscuring the original intent of SBTs—detecting extubation risk under conditions that realistically simulate the post-extubation state.

In our view, this focus on aggregate outcomes has encouraged a trend toward uniform SBT protocols, which may oversimplify the complexity of real-world clinical practice. Patients differ significantly in their cardiopulmonary reserve, risk factors, and the likelihood of receiving post-extubation support such as noninvasive ventilation (NIV). As such, SBT settings should be tailored to the individual patient.

In our ICU, we routinely conduct SBTs using PSV of 5 cmH₂O with PEEP of 5 cmH₂O. While we recognize that this approach is not universally validated, it has proven both physiologically coherent and clinically acceptable in our experience [2]. Crucially, the use of PEEP during SBTs should be informed by the anticipated post-extubation environment. If NIV is expected to be feasible, incorporating PEEP into the SBT provides physiological continuity. On the other hand, when NIV tolerance is uncertain—as in patients with delirium or poor mask fit—removing PEEP during the trial may better unmask latent instability. In patients with reduced ejection fraction, PEEP may support not only respiration but also circulation by reducing cardiac preload and afterload. In those with COPD, it primarily helps maintain airway patency and reduce dynamic hyperinflation. These physiological benefits highlight the need for careful and individualized consideration of PEEP during SBTs.

Rather than seeking a universally optimal SBT setting, we believe the discussion should return to the original goal of SBTs: identifying extubation risk under conditions that faithfully simulate post-extubation physiology. We thank Thille and colleagues for bringing renewed attention to this important topic, and we hope our perspective helps promote a more nuanced and physiologically grounded approach to SBTs.

Sincerely,

Ichita Yamamoto, MD, MHPE.

Tokyo Bay Urayasu Ichikawa Medical Center, Japan.

## Data Availability

Data sharing is not applicable to this article as no datasets were generated or analysed during the current study.
